# First Detected Transmission of C. auris within a Minnesota Healthcare Facility Following Exposure in the Emergency Department

**DOI:** 10.1017/ash.2024.233

**Published:** 2024-09-16

**Authors:** Laura Tourdot, Krista Knowles, Jennifer Dale, Stephanie Homuth, Ginette Dobbins, Tami Dahlquist, Kristy Connors, Jacob Garfin, Jill Fischer, Annastasia Gross, Paula Snippes Vagnone, Ruth Lynfield, Christine Lees

**Affiliations:** Minnesota Department of Health; Minnesota Department of Health Public Health; MN Dept. of Health, Public Health Lab

## Abstract

**Background:** Candida auris reporting and submission of confirmed or possible isolates has been mandatory in Minnesota since August 2019. On August 9, 2023, the Minnesota Department of Health (MDH) was notified of a C. auris isolate in hip tissue from a patient in acute care hospital A (ACH-A). Only 9 cases of C. auris were detected prior to August 2023, in Minnesota, and all from patients with a history of international healthcare or healthcare in endemic C. auris locations of the United States. **Methods:** The MDH Public Health Laboratory (MDH-PHL) confirmed identification of C. auris from the ACH-A isolate by MALDI-TOF. MDH partnered with ACH-A to review medical records, assess infection prevention and control (IPC) practices, conduct contact tracing, and identify patients for colonization screening. Screening was performed on all patients that overlapped with the index case (case A) and were admitted to a facility in the same healthcare system as ACH-A. Facilities accepting discharged patients who overlapped with case A were contacted for colonization screening. Overlapping patients, no longer admitted to a healthcare facility, were sent a notification letter, and offered outpatient screening. Composite axilla/groin swabs were screened for C. auris using real-time PCR at MDH-PHL, who also performed whole genome sequencing (WGS) and single nucleotide polymorphism (SNP) analysis. **Results:** Case A’s medical record showed only Minnesota healthcare exposures, a surgical procedure in June 2023 and indicated the case overlapped with a previous case (case B) from July 2023, who had recent international healthcare. The two cases were hospitalized at ACH-B July 12-18, on different care floors without evident links to shared services. However, the cases were in adjacent rooms in ACH-B Emergency Department (ED) on July 3 for 5 hours, when C. auris status of case B was unknown. WGS indicated both isolates were within clade I (South Asian) and separated by 2 SNPs, suggesting relatedness. Extensive colonization screening occurred among 109 potentially exposed patients, including 18 patients from the ED. No additional C. auris was detected. **Conclusions:** This case represents the first detected transmission of C. auris within a Minnesota healthcare facility. The role of C. auris transmission within the ED is not well understood. Medical record review in combination with WGS analysis suggests potential transmission within the ED. Clinicians should be aware of the risks for C. auris transmission in the ED and follow all IPC measures to prevent transmission of this emerging fungal pathogen.

